# The use of mathematical modeling studies for evidence synthesis and guideline development: A glossary

**DOI:** 10.1002/jrsm.1333

**Published:** 2019-01-08

**Authors:** Teegwendé V. Porgo, Susan L. Norris, Georgia Salanti, Leigh F. Johnson, Julie A. Simpson, Nicola Low, Matthias Egger, Christian L. Althaus

**Affiliations:** ^1^ Population Health and Optimal Health Practices Research Unit, Department of Social and Preventative Medicine, Faculty of Medicine Université Laval Quebec Canada; ^2^ Department of Information, Evidence and Research World Health Organization Geneva Switzerland; ^3^ Institute of Social and Preventive Medicine University of Bern Bern Switzerland; ^4^ Centre for Infectious Disease Epidemiology and Research University of Cape Town Cape Town South Africa; ^5^ Centre for Epidemiology and Biostatistics, Melbourne School of Population and Global Health University of Melbourne Melbourne Australia

**Keywords:** evidence synthesis, glossary, guidelines, knowledge translation, mathematical modeling studies

## Abstract

Mathematical modeling studies are increasingly recognised as an important tool for evidence synthesis and to inform clinical and public health decision‐making, particularly when data from systematic reviews of primary studies do not adequately answer a research question. However, systematic reviewers and guideline developers may struggle with using the results of modeling studies, because, at least in part, of the lack of a common understanding of concepts and terminology between evidence synthesis experts and mathematical modellers. The use of a common terminology for modeling studies across different clinical and epidemiological research fields that span infectious and non‐communicable diseases will help systematic reviewers and guideline developers with the understanding, characterisation, comparison, and use of mathematical modeling studies. This glossary explains key terms used in mathematical modeling studies that are particularly salient to evidence synthesis and knowledge translation in clinical medicine and public health.

## 1 INTRODUCTION

Mathematical models are increasingly used to aid decision making in public health and clinical medicine.^1^, ^2^ The results of mathematical modeling studies can provide evidence when a systematic review of primary studies does not identify sufficient studies to draw conclusions or to support a recommendation in a guideline, or when the studies that are identified do not apply to the specific populations of interest or do not provide data on long‐term follow‐up or on relevant outcomes. For example, mathematical models have been used to inform guideline recommendations about tuberculosis (TB) control in health care facilities,^3^ blood donor suitability with regard to human T‐cell leukemia virus type I (HTLV‐I) infection,^4^ and cancer screening.^5^, ^6^ Mathematical modeling studies are frequently used to synthesize evidence from multiple data sources to address a clinical or public health question not directly addressed by a primary study. For example, a mathematical model was used to synthesize evidence obtained from virological, clinical, epidemiological, and behavioral data to help determine optimal target populations for influenza vaccination programs.^7^ Other examples are mathematical modeling studies that aim to predict the real‐world drug effectiveness from randomized controlled trial (RCT) efficacy data (reviewed in Panayidou et al^7^).

The development of methods for incorporating mathematical modeling studies into evidence syntheses and clinical and public health guidelines is still at an early stage. Systematic reviewers and guideline developers struggle with questions about whether and how to include the results of mathematical modeling studies into a body of evidence. The review of mathematical modeling studies predicting drug‐effectiveness from RCT data identified 12 studies using four different modeling approaches.^7^ Because of the varying use of key terminology between studies, and because certain terms can have different meanings in the literature, it was necessary to describe in the review each modeling approach in detail to illustrate the differences between them. This effort highlights an important reason for the challenges in summarizing the results of mathematical modeling studies. Researchers who develop and analyze mathematical models have different theoretical and practical backgrounds from systematic reviewers, guideline developers, and policy makers, which can result in a lack of a common understanding of concepts and terminology. These communication issues might result either in not using the findings of mathematical modeling studies in evidence synthesis and to inform decision making, or accepting these findings without critical assessment.^8^ A glossary of commonly used terms in mathematical modeling studies that are relevant to evidence synthesis and to clinical and public health guideline development could improve the use of such studies.

A mathematical model is a “mathematical framework representing variables and their interrelationships to describe observed phenomena or predict future events.”^9^ We define a mathematical modeling study as a study that uses mathematical modeling to address specific research questions, for example, the impact of interventions in health care facilities to reduce nosocomial transmission of TB.^10^ For the modeling studies that are most relevant to evidence synthesis and clinical and public health decision‐making, the framework of the mathematical model represents interrelationships among exposure risks, interventions, health outcomes, and health costs (all of these are *variables*) where their interrelationships are typically described by the *parameters* of interest. Mathematical modelers can use different methods to specify these parameters; they can use theoretical values, values reported in the scientific literature, or estimate the parameters from data using methods from statistical modeling. There is some overlap between the terms “mathematical model” and “statistical model” and their uses. Contemporary mathematical modeling studies increasingly include one or more statistical modeling parts. In this glossary, we will consider statistical models as a class of mathematical models that are often integrated into complex mathematical modeling studies to relate the model output to data through a statistical framework.

The goal of this glossary is to provide a common terminology for public health specialists who would like to incorporate the results of mathematical modeling studies in systematic reviews and in the development of guidelines. To identify the terms included in this glossary, we first made an exhaustive list of terms related to mathematical models. Terms were then selected based on discussions among experts attending the World Health Organization's (WHO) consultation on the development of guidance on how to incorporate the results of modeling in WHO guidelines (Geneva, Switzerland, 26 April 2016). Experts included epidemiologists, statisticians, mathematical modelers, and public health specialists. The glossary is divided into three sections. In Section 2, we define some key terms that can be used to characterize the scope of and approach to mathematical models, using examples from the field of infectious disease modeling. In Section 3, we present a list of terms that are commonly used across different research fields in epidemiology to describe more detailed technical properties and aspects of mathematical models. In Section 4, we first discuss how knowledge of the terms can help to assess whether a mathematical modeling study is appropriate for providing evidence for a specific question. We then use the example of the World Health Organization (WHO) guidelines for TB control in health care facilities^3^ to show how mathematical modeling studies can inform recommendations. For more specific definitions of terms that are primarily used in infectious disease modeling, we refer to the glossary by Mishra et al.^11^ Terms appearing in italics are defined in other entries of the glossary.

## 2 TERMS USED TO DEFINE THE SCOPE OF, AND APPROACHES TO MATHEMATICAL MODELS

Before one starts to assess and compare the results of different mathematical modeling studies with each other, it can be helpful to fit them into a larger picture. Experts in systematic reviews and guideline developers need to be able to sort out which modeling studies are likely to help them draw a conclusion, formulate a recommendation, interpret the findings of another study, or understand the clinical or pathological background to a problem. Mathematical modeling studies can be characterized using several dichotomies that help to describe broad aspects, such as the scope and approach. Table 1 provides a list of some important model dichotomies, together with a brief definition, an example, and their relevance to systematic reviews and guideline development.

**Table 1:** Model dichotomies describing the scope of, and approaches to, mathematical models in infectious disease epidemiology

Model Dichotomy^a^	Brief Definition	Example	Potential Relevance or Use for Systematic Review or Guideline Development
Mechanistic vs.	Uses mathematical terms to explicitly describe the mechanisms of infection transmission, pathogenesis and control measures.	*Compartmental model* that describes the transmission of influenza and the effects of vaccination in England and Wales.^12^	Allows implementation and modeling of different vaccination scenarios, such as targeting children or elderly.
Phenomenological	Uses mathematical terms to describe the interrelationships between risks and outcomes without making *assumptions* about the underlying mechanisms.	Estimation and Projection Package (EPP) that fits a simple epidemic curve to HIV surveillance data.^13^	Cannot be used to describe intervention effects in detail, so it is less likely to investigate hypothetical scenarios or interventions.
Predictive vs.	Forecasts future events.	Impact projections of malaria vaccine for timeframes longer than previously conducted trials.^14^	To investigate the expected future impact of implementing or changing interventions, and to set new targets.
Descriptive	Describes and/or explains previously observed henomena.	Quantifying the effect of malaria disease control efforts in Africa between 2000 and 2015.^12^, ^15^	To assess the effectiveness of past interventions or explain previous events and learn from them.
Quantitative vs.	Provides a precise numerical estimation or the expected range of an effect.	HIV prevalence after expanding access to antiretroviral therapy.^16^	To obtain estimates of an effect that can be incorporated into economic (cost‐effectiveness) analyses.
Qualitative	Describes the direction or general size of an effect.	Increasing herpes zoster incidence after mass childhood vaccination against varicella.^17^	Could indicate how and under what conditions an intervention could cause a specific epidemiological outcome. Might influence conditions of a recommendation.
Theory‐driven vs.	Results are driven by theory/assumptions	Investigating the theoretical strategy of universal testing and immediate treatment for HIV.^18^	Can provide a rationale for considering a particular intervention. In the absence of data, results need to be critically evaluated in light of modeling assumptions.
Data‐driven	Results are inferred from data	Influenza transmission model to estimate the effectiveness of historical vaccination programmes.^12^	Can be used to assess effectiveness of interventions where randomised controlled trials are not possible. Evidence primarily relies on the quality of the primary data.

a Some of these dichotomies are adapted from Bolker, 2008.^19^

A fundamental distinction can be made between *mechanistic* and *phenomenological models*. Mechanistic models use mathematical terms to describe the real‐world interactions among different model variables. The parameters governing these models typically have a physical, biological or behavioral interpretation. Infectious disease models, for example, can describe the movement of individuals within hospital wards, and how infections are transmitted upon physical contact between a susceptible and an infected person.^10^ These models have the advantage that specific interventions, such as infection prevention through quarantine or isolation, can be explicitly implemented. Phenomenological models, on the other hand, describe the relationships among different model variables, consistent with fundamental theory, but not derived from first principles. Hence, this type of model does not attempt to describe or explain why and how certain model variables interact, but instead, focuses on the functional relationship that best describes an observed phenomenon. Statistical models, such as regression models, are typically phenomenological and describe the statistical relationship or association between different model variables.

A *predictive model* can forecast future events, such as the course of an epidemic in a given population under different scenarios, whereas a *descriptive model* describes and/or explains previously observed phenomena, such as the effectiveness of past interventions. *Quantitative models* provide a numerical estimation of an intervention effect on model variables, and therefore depend on high‐quality data to inform the model parameters. *Qualitative models* are usually relatively simple models that only provide insights into the direction of an effect, but not its precise magnitude. Nevertheless, they can be used to thoroughly investigate the interrelationships between model variables and the influence of specific parameters on health outcomes (also see *Analytic solution*). Qualitative models can also be useful to explore the potential for unintended consequences of interventions beyond the direct intended effects that might have been observed in RCTs. Finally, an important model dichotomy distinguishes between what drives the results of mathematical modeling studies. Most mathematical models incorporate a combination of some underlying theory, model assumptions, and data. The results of a *theory‐driven* model are primarily based on a priori knowledge or *assumptions* about specific interrelationships, such as the effectiveness of a particular intervention, and are not directly inferred from data. *Data‐driven models* infer their results primarily from data, and are not driven by theory or assumptions that are not well supported.

## 3 TERMS RELATED TO TECHNICAL PROPERTIES AND ASPECTS OF MATHEMATICAL MODELS

### 3.1 Technical terms related to model development and structure

Once the mathematical modeling studies have been broadly characterized, and their purpose has been determined, it is important to gain a better understanding about some of the terms used to describe the technical aspects of the model used in a study. For example, has *heterogeneity* among different individuals been incorporated, or what simulation methods were used to obtain the model results? The following list includes some of the most frequently used terms in mathematical modeling studies in various fields of epidemiology. The terms in Section 3.1 will help in assessing the technical aspects that relate to model development and structure. The terms in Section 3.2 are related to model calibration and validation.

#### 3.1.1 Agent‐based model

See *Individual‐based model*.

#### 3.1.2 Analytic solution

Relates the health outcomes directly to the model parameters using mathematical formulae. Models that can be solved analytically are usually simple models, while more *complex models* typically require a *computational (numerical) solution*.

#### 3.1.3 Assumption

In mathematical modeling studies, assumptions typically relate to the structure of the model and the supposed interrelationships of model variables. An important assumption in infectious disease models concerns the way in which individuals have contacts with each other. This could either be at random or involve some form of heterogeneity. In order to relate the model output to data via a statistical framework, one has to make additional assumptions about the way the data has been gathered and the expected random error.

#### 3.1.4 Compartmental model

This model type stratifies the population into different compartments, such as different health states. Compartments are assumed to represent homogeneous subpopulations within which the entities being modeled–such as individuals or patients–have the same characteristics, for example the same sex, age, risk of infection, or death. The model can account for the transition of entities between compartments (see *State‐transition model*).

#### 3.1.5 Computational (numerical) solution

This describes the approach of solving a mathematical model using either *deterministic* or *stochastic* (see *Monte Carlo methods*) simulation techniques to iteratively calculate the model variables, which are often time‐dependent, for a specific set of parameters. Iteratively calculating the model variables means updating the population characteristics at each time point based on the simulated population characteristics at previous time points. Computational solutions are used when the model is too complicated for deriving an analytic solution.

#### 3.1.6 Continuous‐time model

This is a *dynamic model* where time is treated as a continuous variable (in contrast to a *discrete‐time model*), meaning that the state or value of all other variables (or health outcomes) can be calculated for any time point of interest.

#### 3.1.7 Cycle length

In a discrete‐time model, *cycle length* represents the interval from one time point to the next, for example a specific number of days, weeks, months, or years.^7^

#### 3.1.8 Decision analytic model

This term refers to mathematical models that synthesize available evidence to estimate health outcomes and guide decision making. The term is typically used in health economic analyses.

#### 3.1.9 Deterministic model

This model type typically describes the average behavior of a system (eg, populations or subpopulations) without taking into account stochastic processes or chance events in single entities (eg, individuals). Hence, such models are typically applied to situations with a large number of individuals where stochastic variation becomes less important and heterogeneity can be accounted for using various subpopulations. The parameters of a deterministic model are typically fixed, and a simulation always produces the same result. Deterministic models are typically easier to *calibrate* to data than stochastic models.^11^, ^20^

#### 3.1.10 Discrete‐time model

This type of dynamic model treats time as a discrete variable (in contrast to a *continuous‐time model*) and other variables (or health outcomes) can only change at specific time points.^7^

#### 3.1.11 Dynamic model

A dynamic model contains at least one time‐dependent variable.^11^ This type of model is used to *describe* and *predict* the course of health outcomes (eg, infection incidence) over time when, for example, the exposure risk (eg, infection prevalence) also changes over time.

#### 3.1.12 Heterogeneity

In mathematical modeling studies, this typically describes the differences among individuals, or the variability across parameter values for a specific group of individuals, because of their demographic, biological, or behavioral characteristics.

#### 3.1.13 Individual‐based model

This is a stochastic model representing individuals as discrete entities with unique characteristics. An individual‐based model can be useful to accommodate heterogeneity in a given population. Individual‐based models are also often referred to as *agent‐based* or *micro‐simulation models*. While individual‐based models can provide more realistic representations of a system, they can be difficult to parameterize because they require much more detailed knowledge, or assumptions, of how variables interact. The stochastic nature of these models makes them computationally intensive and challenging to calibrate.

#### 3.1.14 Markov model

A *Markov model* assumes that the future state of variables depends only on the current state, but not the previous states, of variables. For example, in a discrete‐time Markov model, the number of new infected individuals is calculated based on the total number of infected individuals at the previous time step.

#### 3.1.15 Micro‐simulation model

See Individual‐based model.

#### 3.1.16 Monte Carlo methods

These are a class of computational methods that are based on random sampling. Monte Carlo methods are typically used to simulate stochastic models and are computationally intensive.

#### 3.1.17 Ordinary differential equations

Equations that describe the change of a dependent variable, with respect to an independent variable, based on differential calculus. For example, *ordinary differential equations* can be used to describe the increase and decrease of infected individuals in continuous time resulting from acquisition or clearance of infection. Ordinary differential equations are typically used for deterministic and compartmental models.

#### 3.1.18 Parameter

A parameter is a quantity used to describe the interrelationships between model variables. For example, parameters can describe how long different individuals reside in different health states, or how likely they are to transmit a disease to another person. There are different methods to specify the value of parameters. Mathematical modelers can either choose theoretical values based on specific assumptions, or set the values based on literature reviews or model calibration.

#### 3.1.19 Parsimonious model

In a *parsimonious model*, descriptive or predictive, the number of assumptions, parameters and variables is minimized. Parsimonious models are often relatively simple, but they can also become more complex if they achieve the right balance between complexity and explanatory power.

#### 3.1.20 Population‐based model

A type of deterministic or stochastic model where individuals that share the same characteristics, on average, are being grouped into a single population or several subpopulations. In contrast, an individual‐based model treats every individual as a single entity that can have unique characteristics.

#### 3.1.21 State‐transition model

State‐transition models assume that individuals can be in different (health) states and move (transition) between them.^21^ They are typically described using the framework of either Markov models or individual‐based models.

#### 3.1.22 Static model

In a *static model*, all variables are independent of time and constant. A static model typically describes the equilibrium of a system, and relates the model variables for a particular time point only. In contrast to dynamic models, this type of model cannot take into account time‐dependent changes of exposure risks or health outcomes. Decision‐tree models are static models.

#### 3.1.23 Stochastic model

A type of model where the parameters, variables, and/or the change in variables can be described by probability distributions. This type of model can account for process variability by taking into account the random nature of variable interactions, or can accommodate parameter uncertainty, and so may predict a distribution of possible health outcomes. Considering process variability can be particularly important when populations are small or certain events are very rare. Stochastic models are often simulated using Monte Carlo methods.

#### 3.1.24 Time horizon

A *time horizon* denotes a chosen time at which point the effect of an intervention will be evaluated. The time horizon should reflect the health outcomes and the relevant intermediate and long‐term effects of an intervention.^1^

#### 3.1.25 Variable

Variables describe model elements such as exposure risks, interventions, or health outcomes that can vary between settings or over time. The value of a dependent variable (eg, number of infected individuals) changes in relation to an independent variable (eg, time).

### 3.2 Technical terms related to model calibration and validation

#### 3.2.1 Calibration

Calibration is the process of adjusting model parameters, such that the model output is in agreement with the data that are used for model development.^22^ The aim of calibration is to reduce *parameter uncertainty* in order to achieve high model *credibility*.

#### 3.2.2 Credibility

The credibility of a model refers to judgments about the degree to which the model provides trustworthy results. Several dimensions of credibility have been described, including *validity*, design, data analysis, reporting, interpretation, and conflicts of interest.^16^

#### 3.2.3 Sensitivity analysis

A range of techniques used to test the impact of the assumptions made about the parameters. The analysis can be done by changing one parameter (one‐way, univariate), or simultaneously changing several parameters (multi‐way, multivariate). The parameters selected for sensitivity analyses are thought to have an impact on the outcome of interest. In a deterministic sensitivity analysis, a parameter is assigned a limited number of values, while in a probabilistic sensitivity analysis, each parameter is assigned a probability distribution, and parameter values are randomly sampled from these distributions.^1^, ^11^

#### 3.2.4 Uncertainty analysis

A range of techniques to determine the reliability of model results or predictions, accounting for uncertainty in model structure, input parameters, and/or methods used for data analysis.^11^ *Structural uncertainty* relates to the extent to which the structure of the model captures the key features of the system^23^, ^24^, ^25^ and can be analyzed by comparing the results of models with different structures. Parameter uncertainty stems from the model parameters that are used, but whose true values are not known because of measurement error or an absence of evidence.^23^, ^25^ This uncertainty can be analyzed by examining model outputs for a range of values of the parameter. *Methodological uncertainty* arises when there are different methods for analyzing or expressing model outputs. This term is used mostly in health economic modeling.

#### 3.2.5 Validation

A term describing processes for assessing how well a model performs and how applicable the results are to a particular situation.^26^ There are five main types of validation: *face validation* (subjective expert judgment about how well the model represents the problem it addresses); *internal validation (internal consistency, verification,* and addresses whether or not the model behaves as intended and has been implemented correctly); *cross validation* (*convergent validity,* model results are confirmed by other models); *external validation* (model results predict outcomes obtained in a real world setting or in a data set different from the one used for model development); *predictive validation* (model‐predicted events are later corroborated by real‐world observations).^7^, ^27^

## 4 MATHEMATICAL MODELING STUDIES IN GUIDELINE DEVELOPMENT

In addition to providing a useful common terminology for public health specialists and mathematical modelers, the description of different model types and other terms defined in the glossary facilitate interpretation of the results of mathematical modeling studies and inform their incorporation into the guideline development process. As a first step, one needs to identify whether a particular research question, eg, the evaluation of public health programs, long‐term effectiveness or comparative effectiveness, can be investigated using a model. Next, it will be necessary to assess whether existing mathematical modeling studies are appropriate to inform or support a research question or recommendation. We identified four comprehensive frameworks of good modeling practice.^28^ These frameworks cover items such as relevance, conceptualization of the problem, or model structure. Questions such as whether the model population is relevant, the variables represent the desired health outcomes, the necessary heterogeneity is taken into consideration, the time horizon is appropriate, or the assumptions justified can help in the assessment of mathematical modeling studies. Other items concern validity or consistency, ie, the performance of the model according to its specifications. The model should also consider *uncertainty* with regard to the structure, parameters, and methods. Finally, credibility, which takes a number of these items into account, can then be used as the central concept for guideline developers to address the appropriateness of a mathematical modeling study for providing evidence for a specific question,^29^ as illustrated in the following example.

Prevention of TB transmission in hospitals, and particularly of multidrug‐resistant TB, is essential in all countries and requires a combination of strategies. Predicting the spread of TB in a hospital and the surrounding community, and how alternative methods of control might limit the emergence of resistance, are complex nonlinear processes. It is, however, ethically and logistically impossible to conduct RCTs to examine the efficacy of these strategies. Mathematical modeling studies that use observational evidence can therefore play an important role in deciding which strategies are likely to be the most effective. The WHO guideline development group for TB infection control in health care facilities, congregate settings and households assessed systematic reviews of the evidence, which included mathematical modeling studies.^3^

One mathematical modeling study that the guideline committee considered, investigated the effects of several different control measures on the spread of extensively drug resistant (XDR) TB in a community in South Africa.^10^ The model described the transmission of TB in a complex system that included variables representing or contributing to: both the hospital and the surrounding community; different TB health states such as susceptible, latent, infectious, and recovered; drug resistance; HIV infection; and the effects of different control interventions alone and in combination. Hence, the study considered the transmission setting that was of relevance to the guideline, and the model structure included the desired health outcomes and variables. The authors used a mechanistic approach to make explicit the way in which stages in the transmission and natural history of TB are related. A deterministic, *compartmental model*, using ordinary differential equations to describe the transitions between different health states in a dynamic way was appropriate because it allowed the right balance between complexity and tractability. Key parameters that described the natural history, such as rate of natural clearance and rate of relapse, were based on the literature, and their influence was assessed in an uncertainty analysis. Parameters such as the transmissibility coefficient were calibrated using longitudinal data of individuals in a South African community, where data on TB were collected. The model outputs provided quantitative predictions about the percentage reduction in XDR‐TB cases over a reasonable time horizon. External validation of the model was performed using cross‐sectional data with information on the prevalence of TB and of drug resistance and the proportion of resistance cases in people with HIV infection. In summary, the mathematical modeling study covered many of the critical items, and we would conclude that the study has a high credibility.

Compared with natural ventilation, the authors found that mechanical ventilation alone would reduce XDR‐TB cases by 12% (range 10%‐25%). The use of respiratory masks by health workers would prevent 2% of all TB cases, but nearly two‐thirds of XDR cases in hospital staff. The guideline development group considered this study, together with other observational and modeling studies identified through the systematic review. Even though the summarized evidence for the use of ventilation systems and particulate respirators was weak, indirect, and of low quality, the studies suggested that these interventions are favorable for TB infection control.

## CONFLICT OF INTEREST

CA, ME, and NL received funding through a grant from the Special Programme for Research and Training in Tropical Diseases (TDR) to conduct this study. SLN is a member of the GRADE Working Group which develops processes and methods for guideline development. LFJ, TVP, GS, and JAS have no interests to disclose.

## DISCLAIMER

The authors alone are responsible for the views expressed in this article and they do not necessarily represent the views, decisions, or policies of the institutions with which they are affiliated.

## References

[1] Weinstein MC , O'Brien B , Hornberger J , et al. Principles of good practice for decision analytic modeling in health‐care evaluation: report of the ISPOR task force on good research practices–modeling studies. Value Health Jan‐Feb. 2003;6(1):9‐17. 12535234 10.1046/j.1524-4733.2003.00234.x

[2] Easterbrook PJ , Doherty MC , Perriens JH , Barcarolo JL , Hirnschall GO . The role of mathematical modelling in the development of recommendations in the 2013 WHO consolidated antiretroviral therapy guidelines. AIDS Jan. 2014;28(Suppl 1):S85‐S92. 24468950 10.1097/QAD.0000000000000111

[3] World Health Organization . WHO Policy on TB Infection Control in Health‐Care Facilities, Congregate Settings and Households. Geneva: World Health Organization; 2009 9789241598323. 24432438

[4] World Health Organization . Blood Donor Selection: Guidelines on Assessing Donor Suitability for Blood Donation. Geneva: World Health Organization; 2012 9789241548519. 23700651

[5] World Health Organization . WHO Guidelines for Screening and Treatment of Precancerous Lesions for Cervical Cancer Prevention. Geneva: World Health Organization; 2013 9789241548694. 24716265

[6] World Health Organization . WHO Position Paper on Mammography Screening. Geneva: World Health Organization; 2014 9789241507936. 25642524

[7] Panayidou K , Gsteiger S , Egger M , et al. GetReal in mathematical modelling: a review of studies predicting drug effectiveness in the real world. Res Syn Meth Sep. 2016;7(3):264‐277. 10.1002/jrsm.1202 PMC5129568 27529762

[8] Garnett GP . An introduction to mathematical models in sexually transmitted disease epidemiology. Sex Transm Infect Feb. 2002;78(1):7‐12. 11872850 10.1136/sti.78.1.7 PMC1763694

[9] Eykhoff P . System Identification: Parameter and State Estimation. Chichester: Wiley; 1974.

[10] Basu S , Andrews JR , Poolman EM , et al. Prevention of nosocomial transmission of extensively drug‐resistant tuberculosis in rural south african district hospitals: an epidemiological modelling study. Lancet Oct 27. 2007;370(9597):1500‐1507. 17964351 10.1016/S0140-6736(07)61636-5 PMC3711808

[11] Mishra S , Fisman DN , Boily MC . The ABC of terms used in mathematical models of infectious diseases. J Epidemiol Community Health Jan. 2011;65(1):87‐94. 20966445 10.1136/jech.2009.097113

[12] Baguelin M , Flasche S , Camacho A , Demiris N , Miller E , Edmunds WJ . Assessing optimal target populations for influenza vaccination programmes: an evidence synthesis and modelling study. PLoS Med Oct. 2013;10(10):e1001527. 24115913 10.1371/journal.pmed.1001527 PMC3793005

[13] Ghys PD , Brown T , Grassly NC , et al. The UNAIDS estimation and projection package: a software package to estimate and project national HIV epidemics. Sex Transm Infect Aug. 2004;80(Suppl 1):i5‐i9. 10.1136/sti.2004.010199 PMC1765839 15249692

[14] Penny MA , Verity R , Bever CA , et al. Public health impact and cost‐effectiveness of the rts,s/as01 malaria vaccine: a systematic comparison of predictions from four mathematical models. Lancet Jan 23. 2016;387(10016):367‐375. 26549466 10.1016/S0140-6736(15)00725-4 PMC4723722

[15] Bhatt S , Weiss DJ , Cameron E , et al. The effect of malaria control on Plasmodium falciparum in Africa between 2000 and 2015. Nature. Oct 08. 2015;526(7572):207‐211. 26375008 10.1038/nature15535 PMC4820050

[16] Eaton JW , Johnson LF , Salomon JA , et al. HIV treatment as prevention: systematic comparison of mathematical models of the potential impact of antiretroviral therapy on HIV incidence in South Africa. PLoS Med. 2012;9(7):e1001245. 22802730 10.1371/journal.pmed.1001245 PMC3393664

[17] Garnett GP , Grenfell BT . The epidemiology of varicella‐zoster virus infections: the influence of varicella on the prevalence of herpes zoster. Epidemiol Infect Jun. 1992;108(3):513‐528. 1318219 10.1017/s0950268800050019 PMC2272211

[18] Granich RM , Gilks CF , Dye C , De Cock KM , Williams BG . Universal voluntary hiv testing with immediate antiretroviral therapy as a strategy for elimination of hiv transmission: a mathematical model. Lancet Jan 03. 2009;373(9657):48‐57. 19038438 10.1016/S0140-6736(08)61697-9

[19] Bolker BM . Ecological Models and Data in R 2008. Princeton: Princeton University Press; 2008.

[20] Pitman R , Fisman D , Zaric GS , et al. Dynamic transmission modeling: a report of the ISPOR‐SMDM modeling good research practices task force working Group‐5. Med Decis Making Sep‐Oct. 2012;32(5):712‐721. 10.1177/0272989X12454578 22990086

[21] Siebert U , Alagoz O , Bayoumi AM , et al. State‐transition modeling: a report of the ISPOR‐SMDM modeling good research practices task force‐3. Value Health Sep‐Oct. 2012;15(6):812‐820. 10.1016/j.jval.2012.06.014 22999130

[22] Rykiel EJ Jr . Testing ecological models: the meaning of validation. Ecol Model. 1996;90(3):229‐244.

[23] Briggs AH , Weinstein MC , Fenwick EA , et al. Model parameter estimation and uncertainty: a report of the ISPOR‐SMDM modeling good research practices task force‐6. Value Health Sep‐Oct. 2012;15(6):835‐842. 10.1016/j.jval.2012.04.014 22999133

[24] Bilcke J , Beutels P , Brisson M , Jit M . Accounting for methodological, structural, and parameter uncertainty in decision‐analytic models: a practical guide. Med Decis Making. Jul‐Aug 2011;31(4):675‐692. 21653805 10.1177/0272989X11409240

[25] Bojke L , Claxton K , Sculpher M , Palmer S . Characterizing structural uncertainty in decision analytic models: a review and application of methods. Value Health Jul‐Aug. 2009;12(5):739‐749. 10.1111/j.1524-4733.2008.00502.x 19508655

[26] Eddy DM , Hollingworth W , Caro JJ , et al. Model transparency and validation: a report of the ISPOR‐SMDM modeling good research practices task Force‐7. Med Decis Making Sep‐Oct. 2012;32(5):733‐743. 10.1177/0272989X12454579 22990088

[27] Altman DG , Royston P . What Do we Mean by Validating a Prognostic Model. Stat Med Feb 29. 2000;19(4):453‐473. 10694730 10.1002/(sici)1097-0258(20000229)19:4<453::aid-sim350>3.0.co;2-5

[28] Egger M , Johnson L , Althaus C , et al. Developing WHO guidelines: time to formally include evidence from mathematical modelling studies. F1000Res. 2017;6:1584. 29552335 10.12688/f1000research.12367.1 PMC5829466

[29] Jaime Caro J , Eddy DM , Kan H , et al. Questionnaire to assess relevance and credibility of modeling studies for informing health care decision making: an ISPOR‐AMCP‐NPC good practice task force report. Value Health Mar. 2014;17(2):174‐182. 10.1016/j.jval.2014.01.003 24636375

